# Prognostic Cancer Gene Expression Signatures: Current Status and Challenges

**DOI:** 10.3390/cells10030648

**Published:** 2021-03-15

**Authors:** Yuquan Qian, Jimmy Daza, Timo Itzel, Johannes Betge, Tianzuo Zhan, Frederik Marmé, Andreas Teufel

**Affiliations:** 1 Department of Medicine II, University Medical Center Mannheim, Medical Faculty Mannheim, Heidelberg University, 68167 Mannheim, Germany; yuquan.qian@medma.uni-heidelberg.de (Y.Q.); jimmy.daza@medma.uni-heidelberg.de (J.D.); timo.itzel@medma.uni-heidelberg.de (T.I.); Johannes.Betge@umm.de (J.B.); tianzuo.zhan@umm.de (T.Z.); 2 Center for Preventive Medicine and Digital Health Baden-Württemberg (CPDBW), Medical Faculty Mannheim, Heidelberg University, 68167 Mannheim, Germany; 3 Junior Clinical Cooperation Unit Translational Gastrointestinal Oncology and Preclinical Models (B440), German Cancer Research Center (DKFZ), 69120 Heidelberg, Germany; 4 Department of Gynecology, University Medical Center Mannheim, Medical Faculty Mannheim, Heidelberg University, 68167 Mannheim, Germany; frederik.marme@medma.uni-heidelberg.de; 5 Clinical Cooperation Unit Healthy Metabolism, Center for Preventive Medicine and Digital Health Baden-Württemberg (CPDBW), Medical Faculty Mannheim, Heidelberg University, 68167 Mannheim, Germany

**Keywords:** prognostic, gene expression signature, breast cancer, hepatocellular carcinoma, colorectal cancer

## Abstract

Current staging systems of cancer are mainly based on the anatomical extent of disease. They need refinement by biological parameters to improve stratification of patients for tumor therapy or surveillance strategies. Thanks to developments in genomic, transcriptomic, and big-data technologies, we are now able to explore molecular characteristics of tumors in detail and determine their clinical relevance. This has led to numerous prognostic and predictive gene expression signatures that have the potential to establish a classification of tumor subgroups by biological determinants. However, only a few gene signatures have reached the stage of clinical implementation so far. In this review article, we summarize the current status, and present and future challenges of prognostic gene signatures in three relevant cancer entities: breast cancer, colorectal cancer, and hepatocellular carcinoma.

## 1. Introduction

Tumor development involves many genetic alterations, especially in cell growth and proliferation. Since the advent of gene expression analysis, we can detect and classify these changes. By definition, a gene expression signature is a particular group of genes correlating genetic alterations with specific clinical variables, such as diagnosis or prognosis [1]. One of the most important milestones in this area was established in 1995, when the technique known now as microarray was reported to simultaneously analyze expression of multiple genes [2]. Since then, microarrays have revolutionized genetics research and DNA chip technology. The first gene expression signature was presented in 1999, when T.R. Golub et al. used DNA microarray to monitor gene expression of acute leukemia, to produce a classifier that could categorize acute myeloid leukemia (AML) and acute lymphoblastic leukemia (ALL) [3]. From that year on, a plethora of gene expression signatures have been developed and used in various ways, including classifying tumor types, recognizing tumor stages, as well as predicting disease prognosis.

Predicting the prognosis of a disease is crucial for cancer management and is still a challenge for many malignancies due to the limitations of clinicopathologic parameters. Prognostic gene expression signatures can help improve patients’ therapy by classifying tumors into different groups, thus providing guidance for a personalized treatment-decision. The first example was developed in 2002, when Bernards et al. validated the prognostic power of a 70-gene signature in 295 breast cancer (BC) patients by successfully classifying them into good or poor prognosis groups [4]. This test, the first ‘prognostic gene signature’, has been validated in several retrospective studies and finally within a prospective randomized clinical Phase III study, the MINDACT trial [5]. It is marketed as the AGENDIA MammaPrint test and is used in clinical routine practice to guide decisions on the use adjuvant chemotherapy for early breast cancer. Since then, many different prognostic gene expression signatures were produced for literally every organ and tissue [6,7,8,9].

However, despite the significant number of prognostic gene signatures developed, very few have been introduced into clinical routine and even fewer have been thoroughly validated. As breast cancer, colorectal cancer (CRC), and hepatocellular carcinoma (HCC) are ranking among the most common cancers and leading causes of cancer-related deaths on a global scale [10], we aim to summarize the current status of genomic signatures. Furthermore, we note the challenges translating molecular profiles into clinical routine applications and integrate them into clinical decision-making tools to ultimately improve the patients’ treatment and survival and spare unnecessary toxicities.

## 2. Hepatocellular Carcinoma

Fueled by high-throughput technologies, today HCC molecular changes can be investigated in multiple dimensions, including chromosomal structure, genetic polymorphisms, epigenetic changes but also gene expression profiling [11]. Analysis of these gene expression data has produced plenty of HCC prognostic signatures in the last two decades. They were reported to be correlated with clinical outcomes, such as survival [12,13], recurrences [14,15], metastasis [16] and other clinical parameters. Regarding the expression profiling types, most of them are mRNA expression signatures [13,14,15,17], but lncRNA [18], miRNA [19,20] and DNA methylation expression patterns were also reported [21,22]. Due to the broad availability and comparatively low cost, this OMICS technologies were appealing to many scientists around the globe.

The feasibility of using archived specimens, including Formalin-Fixed Paraffin-Embedded (FFPE) samples, in microarray technology to further develop prognostic biomarkers has been demonstrated [12,23]. Most HCC gene signatures are derived from tumor liver tissues, but some are based on adjacent non-tumor tissues [12,24]. In addition to adjacent non-tumoral liver tissues of HCC patients, other liver tissues from non-tumoral patients can also be used for generating HCC prognostic gene signatures. Despite all technological progress some key issues in transcriptomic profiling remain difficult and still halt faster progress in the development of valid gene expression analyzes. Among these critical issues are the considerable heterogeneity of HCC and the problem of diseased control tissue (mostly cirrhotic liver tissue).

Among the first to perform gene expression analysis in HCC, particularly to predict the prognosis of those patients were Iizuka and co-workers in 2003. They analyzed gene expression on 33 HCC quantifying 6000 genes. This first prognostic signature was postulated to predicted early intrahepatic recurrence or nonrecurrence with a positive predictive value of 88% [25]. Throughout the following year several other supervised approaches with varying gene set compositions were published [6,14,15,26].

Since unsupervised signatures failed to be independently confirmed and composition of an ideal signature remained (and remains) undetermined but also due to the technological advance subsequently making full genome expression screens available, unsupervised approaches became increasingly common. Among the first to apply unsupervised approaches were Lee and Thorgeirsson, who published several potentially prognostic signatures [27,28]. In a first publication Lee et al. identified two distinctive subclasses that are highly associated with the survival of the patients providing novel molecular insights into the pathogenesis of HCC [27].

In the following years, gene expression was used to define not just two but more groups in such a way that they could define survival subgroups of hepatocellular carcinoma. Yamashita and co-workers defined 70 EpCAM co-expressed genes to construct a predictive model and define four HCC subgroups: HCC EpCAM positive with either AFP positive or negative and EpCAM negative with either AFP positive or negative. Hoshida et al. performed a meta-analysis to report three distinct HCC subclasses (S1, S2, S3) correlated with different clinical parameters [29]. Meanwhile, six subclasses (G1-G6) were identified by using unsupervised transcriptome analysis [30]. Recently, four prognostic molecular subtypes have been found in Mongolian HCC patients and their comparison with previously reported subclass-related signatures shows similarities and distinctness [31]. Observing the common underlying genomic alterations of different subclasses, Zucman-Rossi et al. classified HCC into two major subgroups, the proliferation and non-proliferation subgroups [32]. Briefly, proliferation HCC is poorly differentiated and is characterized by chromosomal instability and more aggressive clinical features, whereas the non-proliferation subgroup refers to well-differentiated HCCs and shows chromosomal stability and low infiltration potential [33].

Among the most promising signatures, the 5-gene signature established on 314 HCC samples from France used a coefficient and regression formula of a multivariate Cox model to demonstrate an association of HN1, RAN, RAMP3, KRT19, and TAF9 with survival times. These data were further validated in patients from Europe, the United States, and China. The same group furthermore reported a significant predictor of survival in patients treated by resection and ablation and in advanced HCC [34]. However, independent confirmation needs to further accumulate to translate these data into clinical routine decision-making.

In recent years gene expression profiles also became part of larger efforts to integrate genomic, epigenomic, and expression data to reveal prognostic subtypes of HCC. These strategies are highly complex, laborious and certainly need to be proven in independent validation [35].

Besides the above-mentioned HCC signatures, there are also some prognostic signatures of specific subtypes of HCC. Fibrolamellar HCC (FLC) is a rare cancer of the liver that grows in teens and young adults who have healthy livers. Cornella et al. found three molecular classes by using unsupervised clustering, and generated a prognostic 8-gene signature which can predict the survival time of FLC patients [36]. In 2010 Woo et al. found a novel HCC type, cholangiocarcinoma-like HCC, and identified the cholangiocarcinoma-like HCC gene signature which involved in heterogeneous progression of HCC [37]. Moreover, Notch pathway in NASH-driven carcinogenesis was addressed and Notch-active signature are similar in transcription to cholangiocarcinoma-like HCC [38].

Unfortunately, unlike breast cancer, none of the HCC signature-based biomarkers are in routine clinical use. There are many factors accounting for this status quo, mainly involves biological, technological, statistical, and informatics challenges [39]. First, most gene signatures are produced in retrospective studies, and they are more common in sources of bias and confounding. Although these studies can establish an association between gene signatures and HCC prognosis, the results lack reproducibility and cannot enter clinical applications [40]. In addition, validation is important for assessing the accuracy of the predictive power of gene signatures, and it is best to verify in independent patient cohorts. However, Pinyol et al. published in 2019 that 22 previously reported HCC prognostic gene signatures were validated and all of them failed to predict recurrence [41]. Moreover, random gene sets also show a high percentage of the prognostic power of survival for HCC patients [42].

## 3. Colorectal Cancer

A large fraction of CRC is diagnosed in a locally advanced or metastatic stage, therefore prognostic biomarkers are valuable for the selection of specific treatment or surveillance strategies. Several expression signatures have been evaluated in tumor tissues from clinical trials, yielding promising results. However, similar to HCC, multi-gene expression tests are so far not routinely used in the management of CRC [43,44].

Prognostic markers are particularly valuable for Stage II and III CRC, since patients might benefit from adjuvant therapy after surgery. However, the selection of patients is currently suboptimal due to a lack of established biomarkers (except for mismatch repair deficiency), leading to either over- or undertreatment [45]. Differences in tumor biology, reflected by specific expression signatures, might identify patients with particularly favorable prognoses that do or do not require adjuvant chemotherapy. In a recent review, Koncina et al. proposed the following requirements for a powerful signature: (1) Significant prognostic/predictive value in large CRC datasets, (2) homogenous expression within and between tumor tissues, (3) easy clinical translation, (4) independence from clinicopathological features and sample type and (5) analytical and clinical validation [46]. Presently, several expression-based assays with prognostic value for CRC are commercially available and noted in the current NCCN and ESMO guidelines (Table 1) [43,44]. The Oncotype DX test applies a 12 gene expression signature and was used to identify Stage II colon cancer with favorable outcomes in the QUASAR trial [47]. Risk of recurrence at 3 years was 12% versus 22% for low and high recurrence risk groups, respectively, but other histopathological factors such as T stage and mismatch repair status were found to be superior in predicting the risk of recurrence. ColoPrint is another test based on an 18 gene signature [48] that was validated in large retrospective cohorts [49,50], comprising a total of 416 patients with Stage II CRC, of which 124 received adjuvant therapy. Of these patients, 37% were identified as high-risk by ColoPrint. Compared to the low-risk group, 5-year risk of relapse was 21% vs. 10% (HR 2.16, *p* = 0.004). Interestingly, in the subgroup of T3 microsatellite stable tumors, only ColoPrint, but not other clinicopathological features (such as the NCCN guideline risk factors) could predict the risk of relapse. ColDx (commercially distributed as GeneFx Colon) is a prognostic test relying on a 634 gene signature that was tested in several independent cohorts [51,52], including a subsample of 393 patients with Stage II CRC from the Phase III C9581 trial [52]. In this validation cohort, patients classified as high-risk by the ColDx test had a lower 5-year recurrence-free interval (91% vs. 82%; HR 2.13, CI 1.3–3.5; *p* < 0.01). In contrast, only borderline significance was found for age and mismatch repair status. Of note, a high rate of assay failure was observed for this ColDx, which was partly due to the age of the tissue samples. Other gene expression signatures developed for the early-stage CRC include the 5-gene signature Oncodefender [53], the Stage II focused ColoGuideEX [54] and Stage III focused ColoGuidePro [55]. However, none of these signatures were yet validated in an independent cohort or commercialized.

**Table 1:** Commercial gene expression signatures for colorectal cancer.

Gene Signature	Biomarker Sources	Analysis Type	Clinical Outcome	No. Genes	Reference
Oncotype DX Colon	Colon tumor tissue	mRNA	Survival	12	2010, Clark-Langone [56]
ColoPrint	Colon tumor tissue	mRNA	Survival	18	2015, Kopetz [50]
ColDx/GeneFX	Colon tumor tissue	mRNA	Survival	634	2016, Niedzwiecki [52]

Beyond the commercial multi-gene expression tests for early and intermediate-stage colorectal cancer, transcriptome-based analyses have been published in recent years that not only aimed to improve clinical patient stratification, but also to establish new biological classification system of CRC. For instance, reported gene expression signatures specific for adult intestinal stem cells and their derivates in the intestinal crypt, which they established using FACS-purified mouse intestinal stem cells [57]. This classification predicted disease relapse in intermediate-stage CRC patients after resection. Subsequently, several other groups developed molecular classifications of CRC based on profiling of gene expression in tumor samples, thereby aiming to categorize tumors into biologically distinct subgroups that also differed in prognosis and response to targeted therapies. [58,59,60,61,62,63]. All those classifications identified mismatch repair-deficient tumors as a distinct subtype, but apart from that, they were largely different. To resolve those inconsistencies, an international consortium was formed, aiming to unify the different classification systems by identifying core subtype patterns and ultimately facilitating better clinical translation [64]. This collaborative effort led to the development of consensus molecular subtypes (CMS), a transcriptome-based classification categorizing CRCs into four subtypes with distinct tumor biology: CMS1 (mismatch repair deficiency/immune type) CMS2 (canonical type), CMS3 (metabolic type), and CMS4 (mesenchymal type) [64]. Interestingly, the subtypes could be related to previous genomic, epigenetic, histological and clinical features of CRC [64,65]. For instance, CMS1 was found to be largely overlapping with mismatch repair deficient/hypermutated cancers that are also associated with hypermethylation, a higher frequency of BRAF mutations, location in the proximal colon, low tumor differentiation, and a high degree of immune cell infiltration. In contrast to that, CMS2 and CMS4 were associated with a high degree of copy number variations, mismatch repair deficiency and a low degree of methylation. CMS2, representing the “canonical subtype” was further associated with WNT and MYC activation, as well as a left-sided location, while CMS4 was related to mesenchymal signatures, TGF-beta activation, angiogenesis pathways, and extracellular matrix remodeling, histologically characterized by a strong desmoplastic reaction [64,65]. Lastly, CMS3 was found to have mixed microsatellite instability (MSI) status, low copy number, and methylation status and, as the most prominent feature of this subtype, marked metabolic reprogramming, including activation of glutaminolysis and lipidogenesis [64,65,66].

With respect to prognosis, CMS4 was associated with the worst overall survival and relapse-free survival in early and intermediate-stage cancer, while CMS1 cancers showed the worst survival rates after relapse [64]. Additionally, the value of CMS as a prognostic marker for intermediate-Stage II and III CRC was assessed by two further studies. In the study of Song et al., 1729 patients who received fluoropyrimidine and oxaliplatin were assessed by different transcriptome-based classifiers, including CMS. Here, CMS4 predicted poor outcome in Stage II/III cancers [67]. In contrast, another study applied several different classifiers on a total of 2636 patients with Stage II/III CRC who were either treated or not treated with adjuvant chemotherapy [68]. Results from this large study revealed that only clinicopathological features and a high cytotoxic lymphocytes infiltration (which is represented by the CytoLym score) was associated with improved disease-free survival, while no association was observed for CMS subtypes. Despite the evidence favoring the use of selected prognostic signatures, routine use of these expression-based tests is not recommended for intermediate-stage CRC since evidence from prospective trials assessing their value for predicting benefit from chemotherapy is lacking and only minor prognostic differentiation margins were observed. However, their use can be considered for complementing clinicopathological information in specific scenarios with intermediate-risk cancer [44].

In metastatic disease, the impact of CMS is different than in intermediate stages. First, CMS1 (not CMS4) is associated with the worst prognosis in this stage, while CMS2-4 have better overall outcomes [69,70], a finding that is corresponding to the data from mismatch repair deficient cancers with favorable prognosis in early stages and worse prognosis in Stage IV disease [71]. CMS has also been studied as a predictive signature for therapy selection in metastatic cancers, an important research topic since only a few markers including mismatch repair deficiency, RAS/RAF status and tumor sidedness are currently available for this purpose [46]. To this end, CMS1 is most likely associated with sensitivity to immune checkpoint inhibitor treatment due to its association with MSI/hypermutation [72], although data from clinical trials in this regard are not yet available. With respect to prediction of outcome upon treatment with EGFR and VEGF antibody combination therapies, published data are in part conflicting. The Phase III FIRE-3 and CALGB/SWOG 80405 clinical trials compared the addition of bevacizumab or cetuximab to 5-FU-based doublet chemotherapy as first-line treatment of advanced CRC and retrospective transcriptomic analysis for CMS classification of tumors from these trials have been performed. In the CALGB/SWOG 80405 analysis, CMS1 patients treated with bevacizumab had better overall survival (OS) than those treated with cetuximab, while the CMS2 patients benefited more from cetuximab therapy. According to the FIRE-3 study data, OS was comparable in CMS1 and CMS2 subgroups independent of targeted therapy, while CSM3 and CSM4 both favored cetuximab with a longer OS. Additionally, retrospectively analyzed data from the AGITG MAX trial suggested CMS2 and possibly CMS3 tumors benefit from bevacizumab in addition to capecitabine chemotherapy, compared with other CMS [73]. Other retrospective data suggested a worse outcome associated with anti-EGFR antibodies in CMS1 and a favorable outcome in CMS2 [74]. Differences of CMS predictive values found in these studies have been attributed to different chemotherapy backbones used in the trial populations, the interaction of those chemotherapies with targeted therapies and the tumor microenvironment and differences in therapy sequence [75]. Interestingly, in a different approach classifying molecular subtypes based on gene copy number variations instead of gene expression [76] found that tumors with a high or intermediate degree of chromosomal instability had improved outcome after bevacizumab combination therapy, while the subgroup with a low degree of copy number variations (corresponding CMS1 or mismatch repair deficient/hypermutated tumors) did not benefit from bevacizumab treatment. With respect to conventional chemotherapies, the improved outcome of irinotecan- vs. oxaliplatin-based combination treatment has been reported for CMS4 tumors [74]. Thus, summarizing these data, cetuximab might be beneficial in CMS2-4 tumors and irinotecan specifically in CMS4 tumors, while the situation for bevacizumab seems less clear. Further data, favorably from prospective trials, are needed to analyze the predictive value of CMS in metastatic CRC. Of note, studies in preclinical models of CRC revealed strong associations of CMS with the response to specific anticancer drugs, including oxaliplatin [77], HSP-90 inhibitors [78], the SMAC-mimetic birinapant [79] or the surviving repressor YM-155 [80], indicating that the CMS classification has predictive potential for specific substances.

Thus, CMS represents a transcriptome-based classifier of CRC that has prognostic value in both intermediate and advanced stages of CRC. It also appears to have predictive value for specific drugs, although associations of CMS with outcome upon treatment with currently used combination therapies have been difficult and in part conflicting, demanding prospective trials to determine the true value of this classification in clinical practice.

In summary, CMS subtyping of CRC is not routinely used in clinical practice, lacks standardization, and requires bioinformatics resources, which currently limits a more widespread use. On the other hand, commercially available and more easy-to-use prognostic expression-panels for early and intermediate-stage prognostication lack a clear biological interpretability, have no predictive value, and should only be used in specific clinical situations.

## 4. Breast Cancer

Breast cancer is, to date, the most successful field regarding the clinical use of validated multi-gene prognostic tools, and the impact of genomic panels in BC management is noticeable. In the 8th cancer staging manual by the American Joint Committee on Cancer 2017 update, 5 multi-gene panels (Oncotype DX, MammaPrint, EndoPredict, Prosigna/PAM50, and the Breast Cancer Index; Table 2) are considered to be stage modifiers, reclassifying low-risk hormone receptor-positive, HER2-negative, and lymph node-negative tumors of any size into Stage I, the same prognostic category as T1a-T1bN0M0 [81]. In the following paragraphs, we will describe the most relevant prognostic gene signatures to date.

**Table 2:** Summary of breast cancer commercially available gene expression signatures.

Gene Signature	Biomarker Sources	Analysis Type	Clinical Outcome	No. Genes	Reference
Oncotype DX Breast	Breast tumor tissue	mRNA	Survival, benefit of chemotherapy	21	2004 Paik [82]
Mammaprint	Breast tumor tissue	mRNA	Survival	70	2002 van’t Veer [83]
Endopredict	Breast tumor tissue	mRNA	Survival	12	2017 Warf [84]
Prosigna/PAM50	Breast tumor tissue	mRNA	Survival	50	2009 Parker [85]
Breast Cancer Index	Breast tumor tissue	mRNA	Survival, benefit of hormone therapy after 5 years	7	2008 Ma, 2013 Sgroi [86,87]

During the early 2000’s, the development of gene expression profiling tests allowed for multiple gene expression signatures in breast cancer to be established empirically by comparing gene expression data from patients who did or did not presented recurrences [88]. These tests identified ER-related genes and proliferation markers as the two most powerful molecular processes to predict an initial clinical outcome (i.e., the first 5-years) but are not able to predict late recurrences, a relatively common issue in ER-positive breast cancer [89]. Only two first-generation tests are commercially available: Oncotype DX and MammaPrint.

### 4.1. Oncotype DX

Oncotype DX (Genomic Health, Redwood, CA, USA) uses RNA isolation of FFPE breast cancer tissue followed by RT-PCR. It analyses a 21-gene signature, 16 cancer-related and 5 reference genes (Table S1), selected in a “candidate gene” approach according to their known relevance in breast cancer [82], and provides a “Recurrence score” (RS) that categorizes the patients into three recurrence risk groups (low/intermediate/high) [90]. The RS prognostic value was first retrospectively evaluated in a series of 447 node-negative breast cancer patients, who received adjuvant Tamoxifen only within the NSABP B14 protocol [82], and in node-positive patients from the TransATAC [91] study. Its potential to predict benefit from adjuvant chemotherapy has been retrospectively explored with samples from node-negative patients in NSABP B-20 and node-positive patients in SWOG 8814 [92]. Recently, TAILORx, a Phase III study prospectively addressed the question which patients with HR-positive and HER2-negative, node-negative breast cancer benefited from adjuvant chemotherapy. For this purpose, the cut-offs between the risk groups were modified and patients with a RS of <11 were regarded as low-risk and did not receive adjuvant chemotherapy, whereas patients with an RS of greater than 25 were considered high-risk and were all recommended chemotherapy. The remaining group with a Recurrence Score ranging from 11 to 25 were randomly assigned to receive adjuvant chemotherapy or observation in addition to standard of care endocrine therapy. In the overall study population, no benefit from adjuvant chemotherapy was observed. Only in patients with an age of 50 years and below a small benefit from adjuvant chemotherapy was suggested above an RS of 15. There is an ongoing debate as to whether these effects were mainly due to endocrine effects of chemotherapy [90]. Very recently, data from the RxPONDER trial (ClinicalTrials.gov, ID: NCT01272037, Access date: 1 February 2021), investigating the predictive potential of Oncotype DX RS in patients with HR-positive, HER2-negative breast cancer and 1 to 3 involved axillary lymph nodes were presented [93]. Here, all patients with a RS between 0 and 25 were randomized to adjuvant chemotherapy and endocrine therapy or endocrine therapy alone. In this node-positive population RS did not predict the benefit from chemotherapy. Again, similar to what was observed in TailorX, premenopausal patients did experience a small absolute benefit from chemotherapy irrespective of their RS. From this data it has become clear that post-menopausal patients with 1 to 3 involved lymph nodes can safely be spared adjuvant chemotherapy. In premenopausal patients, this decision needs individual discussion. 

Therefore, the Oncotype DX RS delivers important prognostic information but also predictive information on the benefit from adjuvant chemotherapy or the lack thereof in HR-positive, HER2-negative early invasive breast cancer with up to three involved axillary lymph nodes [92]. It is currently recommended in clinical guidelines with the highest level of evidence, including the NCCN algorithm with a level of evidence 1 for both predictive and prognostic power in ER+, HER2- and node-negative patients and 2A for node-positive patients [94], the American Society of Clinical Oncology (ASCO) [95], ESMO [96], the UK NICE Diagnostic Guidance [97] and the German Gynecological Oncology working group (AGO) [98].

### 4.2. Mammaprint/Blueprint

MammaPrint (Agendia, Amsterdam, The Netherlands) requires FFPE or fresh breast cancer tissue to perform an RNA microarray analysis. Its 70-gene signature (Table S1) is based on the Amsterdam Breast cancer signature, a gene list developed after the analysis of global gene expression profiles of 78 tumor samples in a statistical “top-down” approach regarding their relation with early recurrence in untreated node-negative breast cancer patients, and the prognosis was initially validated in a series of 295 node-negative and node-positive breast cancer patients [83]. In the TRANSBIG consortium study, the 70-gene signature showed a better stratification of high and low-risk patients when compared to the Adjuvant! Online tool [99]. Later, it was prospectively validated y as a prognostic tool for early-stage ER-positive and HER2-negative node-negative breast cancer in the observational RASTER study [5,100].

Finally, the prospective randomized Phase III MINDACT study investigated if patients considered to be clinically high-risk of recurrence as defined by the Adjuvant! Online tool but low genomic risk according to MammaPrint, could safely forego adjuvant chemotherapy. The study included 6693 women with early breast cancer and up to 3 metastatic axillary lymph nodes, and showed that chemotherapy could be avoided in such patients [5].

However, unlike Oncotype DX, it has not shown an ability to directly determine the benefits of CT in any of its risk groups [90]. MammaPrint can be used to guide clinical decisions and is included in the majority of BC clinical guidelines with the highest level of evidence, including the NCCN algorithm with a level of evidence 1 as a prognostic tool in HR-positive, HER2-negative breast cancer with up to 3 positive nodes [94], ASCO [95], ESMO [96] and AGO [98]. However, it is not recommended by the NICE guidelines since the cost-effectiveness was considered insufficient [97].

To date, three commercially available signatures (Prosigna, Endopredict, Breast Cancer Index) are recommended by several guidelines as prognostic tools in HR_positive, HER2-negative breast cancer with up to 3 positive nodes, including the NCCN algorithm with a level of evidence 2A [94], the ASCO guidelines (moderate strength) [95] and the ESMO guidelines (1B) [96], and AGO guidelines (AGO+) [98]. It is worth noting that both Prosigna and the Breast Cancer Index (BCI) address the risk of relapse after the first 5 years of endocrine therapy [94,96,101].

### 4.3. Endopredict

Endopredict (Myriad Genetics, Salt Lake City, UT, USA) is a 12-gene signature (Table S1) that uses RNA extracted from FFPE breast cancer tissue for RT-PCR to determine the expression of eight cancer genes, three RNA reference genes for normalization, and one DNA control gene to assess for DNA contamination [84]. The commercially available test, EPclin, considers clinical factors such as tumor stage and nodal status in the algorithm and provides a binary result to determine whether adjuvant chemotherapy should be recommended and informs about the risk of late recurrences (EPclin low-risk < 3.3, EPclin high-risk ≥ 3.3). EPclin, and was clinically validated in the ABCSG6 and ABCSG8 randomized Phase III trials, which included over 1700 post-menopausal BC patients treated with endocrine therapy alone, demonstrating that EP is prognostic for early (years 1–5) and late (years 5–10) distant recurrences in node-negative and node-positive disease [102]. EPclin is supported in the most recent AGO, ESMO and NCCN guidelines as a prognostic tool for both treatment decisions and was showed to outperform Oncotype DX RS score in distant recurrence prediction [103].

### 4.4. Prosigna/PAM50

Prosigna/PAM50 (NanoString Technologies, Seattle, WA, USA) is a 50 genes signature (Table S1) that considers both the PAM50 genetic profile, derived with qRT-PCR from FFPE breast cancer tissues [85], with clinicopathological features as tumor size and nodal stage and a proliferation signature, providing a Risk of Recurrence score (ROR) that stratifies the tumor into three 10-year distant Recurrence score categories: low (<10%), intermediate (10–20%) and high (>20%) and, similar to the MammaPrint/BluePrint test, it provides molecular subtype information on the tumor [104].

Its use as a prognostic tool was validated in the ABCG8 [104] and TransATAC trials [91]. It is supported by current guidelines (ESMO, AGO, NCCN) to guide the use of adjuvant chemotherapy and is also prognostic for late recurrences [103].

### 4.5. Breast Cancer Index (BCI)

The Breast Cancer Index (BCI, bioTheranostics, San Diego, CA, USA) analyses 7 genes (Table S1), 5 known proliferation genes called Molecular Grade Index (MGI) and the ratio between HOXB13: IL17BR, to assess the need for extended hormone therapy [86,87]. It is currently the only multi-gene test that functions as a predictive biomarker of late recurrence (i.e., beyond 5 years) and directly addresses the question of whether the patient would benefit from extended endocrine therapy in early-stage, HR+ breast cancer, aiming to avoid overtreatment with extended hormonal. The signature has been retrospectively validated on samples from the ATAC, the aTTom as well as the IDEAL trials [105] and is now recommended for this use in the 2021 NCCN guideline [106]. There are some very preliminary data to suggest that BCI might also be of used to predict benefit from extended endocrine therapy in the HER2-positive subset of HR-positive patients, but further studies are required [107].

### 4.6. Other BC Gene Signatures

Fewer studies have focused on triple-negative (TNBC) or HER2-positive disease, presumably because the indication for adjuvant chemotherapy is less uncertain in most of these cases. However, several gene expression studies on TNBC, have helped to increase the understanding of this heterogenous disease, which is not characterized by common features but rather by the common absence of the three markers. Initially, Lehmann et al. identified six biologically distinct subgroups within triple-negative breast cancer, basal-like 1, basal-like 2, luminal androgen receptor (LAR), mesenchymal, mesenchymal stem-like and immunomodulatory subtypes [108]. The classification, also known as the Vanderbilt classification, has later been simplified [109] based on additional data and the subclasses have been reduced to 4 distinct subtypes, namely basal-like 1 & 2, LAR and the mesenchymal subtype. Independent groups have defined simar subclasses in TNBC [110,111,112,113], Although these signatures have led to a new subclassification and greatly improved our understanding of this disease, they have not found a clinically useful application yet. However, trials are ongoing in, for example, the LAR subtype to investigate the use of anti-androgens.

### 4.7. Clinical Use of Genomic Signatures in Breast Cancer

Currently, the commercially available BC genomic signatures help clinicians define the treatment in two specific clinical situations: supporting adjuvant chemotherapy in early ER+ HER2− BC patients and supporting adjuvant extended hormone therapy in post-menopausal ER+ BC patients.

#### 4.7.1. Supporting Adjuvant Chemotherapy in Early ER+ HER2− BC Patients

In early-stage estrogen-receptor positive and HER2 negative breast cancer patients with node-negative disease or with up to 3 positive lymph nodes, the use of the 21-gene Recurrence Score (Oncotype DX) is currently recommended to predict the benefit of adjuvant chemotherapy and is the only test that has been prospectively validated for this purpose. Patients 50 years of age or older with HR-positive, HER2-negative disease, and 0–3 involved lymph nodes can safely be spared adjuvant chemotherapy. In patients younger than 50 year with node-negative disease and a RS of >15, a small benefit from chemotherapy cannot be excluded and need a discussion on an individual basis. Patients younger than 50 with 1 to 3 involved lymph nodes should be offered adjuvant chemotherapy as they derive a small but statistically significant benefit regardless of the Recurrence score. Adjuvant chemotherapy should also be offered to all patients with a RS of >25. The 70-gene signature (MammaPrint) can also help to identify patients who are deemed at high clinical risk but are classified as genomic low-risk who have been demonstrated to have a favorable prognosis even without chemotherapy. Other multi-gene signatures offer important prognostic information and if they predict a low ROR are used to select patients for adjuvant endocrine therapy alone in routine clinical practice and based on guidelines, even though they have strictly not prospectively been demonstrated to predict the benefit of adjuvant chemotherapy.

#### 4.7.2. Supporting Adjuvant Extended Hormone Therapy in Post-Menopausal ER+ BC Patients

Prosigna, BCI, and EPclin have been demonstrated to predict late recurrences in patients with HR-positive, HER2-negative breast cancer after 5 years of endocrine therapy. BCI has also demonstrated to predict the benefit of extended endocrine therapy in the retrospective analysis of two clinical trials addressing this question (aTTom, IDEAL) and is the only test recommended specifically for this purpose.

However, these two questions are rarely regarded independently, so in clinical routine testing mostly is initiated after surgery to help the decision about adjuvant chemotherapy. These results will then influence the decision about extended endocrine therapy in addition to clinicopathologic features (tumor size, lymph node status etc.) Oncotype DX and MammaPrint have not provided similar evidence to predict late recurrences. In the case of MammaPrint, this is quite comprehensible, as it was specifically trained to identify patients with recurrences during the first 5 years after diagnosis. With respect to late recurrences, BCI offers the highest level of evidence [103].

## 5. Discussion and Conclusions

The development of high-throughput gene expression testing, addressing relevant clinical questions, has allowed an optimized use of the current therapeutic resources by reducing the rates of over/under treatment and avoiding unnecessary side-effects in cancer patients. To date, the field of breast cancer research has a lead in the area. Due to several factors including the significant interest in the disease, high incidence, mortality, and social burden, the identification of different biomarkers robustly linked to the patient’s prognosis and treatment response, and the identification of different molecular subtypes of the disease, each one with clearly distinguished medical treatments, was rapidly progressing. However, there remain open questions, and there is still some debate about the use of gene signatures in early BC with positive lymph nodes. The rXPONDER trial has recently answered this important aspect, but still reimbursement remains an issue in the setting and these signatures are globally not accessible to all patients, despite the fact that their use to avoid chemotherapy appears to be cost-effective [114,115] and importantly avoids long term toxicities [116].

Hepatocellular carcinoma remains probably the most challenging entity. Arising mostly on the cirrhotic liver, comparison of diseased samples against non-tumorous tissue is difficult as the control tissue itself may have significant changes in gene expression. The analysis is even more complicated by a clearly heterogeneous tumor entity. Against this background, algorithms were developed that using a large number of random signatures and applying “swarm intelligence” [42] but also correlation across multiple tumor samples [117]. However, these novel concepts also need further independent validation. Potential progress may arise from focusing on a specific highly prevalent entity such as fatty liver disease or chronic hepatitis B to characterize a better-defined subgroup and control tissue.

In CRC, the classification in CMS partly using transcriptomic profiles also remains difficult. CMS were well evaluated in CRC studying two comparable Phase III trials. However, these studies led to completely different results. Difficulties here may be to classify all patient samples in one of four subtypes, characterized by multiple molecular features. Some samples may have mixed results characteristics which may not fit easily in one of the proposed categories. Instead of forcing those samples into one specific subgroup it may be more beneficial to the overall result to exclude some samples which are not easily classifiable. Although this will result in no prognosis prediction for some patients, CMS classification effectiveness and quality of the prediction for the remaining samples may improve and be more robust.

Potential solutions for improving the gene expression analysis in particular hepatocellular carcinoma but also CRC and BC will certainly arise from a better definition of standard procedures for sample collection and preservation but also from standardized use of the diagnostic algorithms leading to the classification in gene expression analysis. Various publications have demonstrated the potential of integrative OMICS analyzes incorporating different biological levels in which the transcriptome may play a valuable role. This appearance of new integrative tools including multi-omics analysis could push the field forward and improve the clinical outcomes in these devastating diseases.

## Supplementary Materials

The following are available online at https://www.mdpi.com/2073-4409/10/3/648/s1, Table S1: Genes analyzed in the commercially available breast cancer expression signatures.
