# Supplementary material for: Prognostic Cancer Gene Expression Signatures: Current Status and Challenges
Source: Cells. 2021 Mar 15;10(3):648. doi: 10.3390/cells10030648 (PMC8000474; doi:10.3390/cells10030648)
Supplement: Supplementary file 1 [file cells-10-00648-s001.pdf]

Table S1. Genes analyzed in the commercially available breast cancer expression signatures.

Oncotype DX [82]	Mammaprint [83]	Endopredict [84]	Prosigna/PAM50 [85]	Breast Cancer Index [86,87]
**Proliferation genes**	AL080059	**Genes of interest**	ACTR3B	**Molecular grade index**
Ki67	Contig63649_RC	BIRC5	ANLN	**(MGI)**
STK15	Contig46218_RC	UBE2C	BAG1	BUB1B
Survivin	LOC51203	DHCR7	BCL2	CENPA
CCNB1 (Cyclin B1)	AA555029_RC	RBBP8	BIRC5	NEK2
MYBL2	ALDH4	IL6ST	BLVRA	RACGAP1
	Contig38288_RC	AZGP1	CCNB1	RRM2
**Invasion genes**	FGF18	MGP	CCNE1	
MMP11 (Stromolysin	Contig28552_RC	STC2	CDC20	**Ratio between:**
3)	Contig32125_RC		CDC6	HOXB13
CTSL2 (Cathepsin L2)	BBC3	**Normalization Genes**	CDH3	IL17BR
	AL137718	CALM2	CENPF	
**HER2 genes**	KIAA1442	OAZ1	CEP55	
GRB2	DC13	RPL37A	CXXC5	
HER2	CEGP1		EGFR	
	EXT1	**Control Gene**	ERBB2	
**Estrogen genes**	FLT1	HBB	ESR1	
ER	GNAZ		EXO1	
PGR	OXCT		FGFR4	
BCL2	MMP9		FOXA1	
SCUBE2	Contig55377_RC		FOXC1	
	Contig35251_RC		GPR160	
**Other genes**	ECT2		GRB7	
GSTM1	GMPS		KIF2C	
CD68	HEC		KRT14	
BAG1	WISP1		KRT17	
	PK428		KRT5	
**Reference genes**	SERF1A		MAPT	
ACTB (b-actin)	FLJ12443		MDM2	
GAPDH	GSTM3		MELK	
RPLPO	Contig32185_RC		MIA	
GUS	RAB6B		MKI67	
TFRC	Contig48328_RC		MLPH	
	Contig46223_RC		MMP11	
	UCH37		MYBL2	
	PECI		MYC	
	AK000745		NAT1	
	Contig40831_RC		NDC80	
	TGFB3		NUF2	
	KIAA0175		ORC6L	
	COL4A2		PGR	
	L2DTL		PHGDH	
	HSA250839		PTTG1	
	DCK		RRM2	
	FLJ22477		SFRP1	
	DKFZP564D0462		SLC39A6	
	SLC2A3		TMEM45B	
	AF257175		TYMS	
	ORC6L			

	RFC4	UBE2C
	Contig55725_RC	UBE2T
	Contig24252_RC	
	CFFM4	
	MCM6	
	AP2B1	
	TMEFF1	
	IGFBP5	
	LOC57110	
	MP1	
	IGFBP5	
	NMU	
	AKAP2	
	FLJ11354	
	PRC1	
	Contig20217_RC	
	CENPA	
	SM-20	
	CCNE2	
	ESM1	
	FLJ11190	
